# Effects of in-office bleaching agent combined with different desensitizing agents on enamel

**DOI:** 10.1590/1678-7757-2018-0233

**Published:** 2018-11-08

**Authors:** Zeynep B. Kutuk, Esra Ergin, Filiz Y Cakir, Sevil Gurgan

**Affiliations:** 1 Hacettepe University, Department of Restorative Dentistry, Ankara, Turkey

**Keywords:** Tooth bleaching, Desensitizing agents, Color, Hardness test, Chemical analyses

## Abstract

**Objective:**

To analyze color change, microhardness and chemical composition of enamel bleached with in-office bleaching agent with different desensitizing application protocols.

**Materials and Methods:**

One hundred and seventeen polished anterior human enamel surfaces were obtained and randomly divided into nine groups (n = 13). After recording initial color, microhardness and chemical composition, the bleaching treatments were performed as G1: Signal Professional White Now POWDER&LIQUID FAST 38% Hydrogen peroxide(S); G2: S+Flor Opal/0.5% fluoride ion(F); G3: S+GC Tooth Mousse/Casein Phosphopeptide-Amorphous Calcium Phosphate (CPP-ACP) paste(TM); G4: S+UltraEZ/3% potassium nitrate&0.11% fluoride(U); G5: S+Signal Professional SENSITIVE PHASE 1/30% Nano-Hydroxyapatite (n-HAP) suspension(SP); G6: S-F mixture; G7: S-TM mixture; G8: S-U mixture; G9: S-SP mixture. Color, microhardness and chemical composition measurements were repeated after 1 and 14 days. The percentage of microhardness loss (PML) was calculated 1 and 14 days after bleaching. Data were analyzed with ANOVA, Welch ANOVA, Tukey and Dunnett T3 tests (p<0.05).

**Results:**

Color change was observed in all groups. The highest ΔE was observed at G7 after 1 day, and ΔE at G8 was the highest after 14 days (p<0.05). A decrease in microhardness was observed in all groups except G6 and G7 after 1 day. The microhardness of all groups increased after 14 days in comparison with 1 day after bleaching (p>0.05). PML was observed in all groups except G6 and G7 after bleaching and none of the groups showed PML after 14 days. No significant changes were observed after bleaching at Ca and P levels and Ca/P ratios at 1 or 14 days after bleaching (p>0.05). F mass increased only in G2 and G6, 1 day after bleaching (p<0.05).

**Conclusions:**

The use of desensitizing agents containing fluoride, CPP-ACP, potassium nitrate or n-HAP after in-office bleaching or mixed in bleaching agent did not inhibit the bleaching effect. However, they all recovered microhardness of enamel 14 days after in-office bleaching.

## Introduction

Esthetics is a major concern in dentistry today. Changes in the smile has surprising effects on the self-esteem of an individual, especially in a society that places too much emphasis on physical appearance. ^1^ Tooth bleaching is one of the most requested esthetic procedures asked for by patients who want an attractive smile. Today, many different bleaching systems have been introduced to meet this demand. ^2^ Although the at-home bleaching system using 10% carbamide peroxide was considered the standard treatment for vital teeth in the past, in-office technique has become more popular than at-home bleaching, as highly concentrated (30%-35% hydrogen peroxide) products promote faster tooth whitening. ^3^ Regardless of the technique or product used, the mechanism of action of bleaching agents is based on a complex oxidation process with release of reactive oxygen species, which penetrate through the pores of enamel rods and reach the dentin, breaking down organic molecules and producing lighter, smaller, and clearer compounds. ^4^

In-office bleaching has been validated as effective for lightening tooth color, most clinical studies have shown that more than 70% of the patients who undergo in-office bleaching complain of tooth sensitivity and this is the main deterrent for patients to successfully complete their bleaching treatment. ^5^ Some remineralizing components, such as fluoride, calcium, amorphous calcium phosphate, and hydroxyapatite are used to minimize adverse effects of bleaching treatments on the enamel. ^6^ In an attempt to decrease or limit dental sensitivity during bleaching, a number of different desensitizing agents have been introduced for use before or after bleaching or in association with bleaching gels. ^7^ These components are added in the bleaching gel to prevent demineralization of the enamel during bleaching and the decrease in dental sensitivity reported by many patients during and after bleaching treatment. ^8^ However, there are conflicting reports about the effects of bleaching agents on alterations of the surface morphology and chemical properties of dental tissues. ^9^

Studies comparing the effect of desensitizing agents on the potential of bleaching gels and on the enamel structure are limited.^6,10,11^ Therefore, this study aimed to evaluate the effects of different desensitizing protocols on the efficacy of in-office bleaching agent and microhardness and chemical composition of human enamel *in vitro.* The null hypothesis tested was that the use of desensitizing agents with the bleaching system would alter the bleaching efficacy and would have no beneficial effect on enamel.

## Materials and Methods

This study was conducted in accordance with all the provisions of the local human subjects oversight committee, with the Declaration of Helsinki (1964) and with the policies of Hacettepe University. The approval code for this study is FON 12/19.

**Figure 1:**
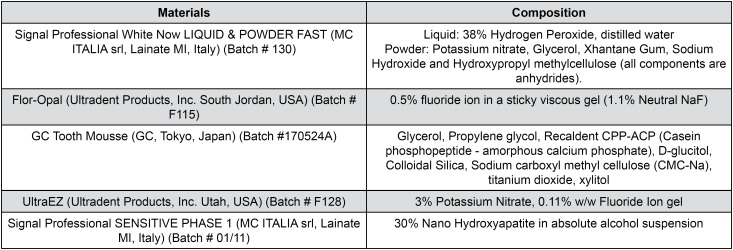
Materials used in the study

Figure 1 shows the materials and their compositions used in the study.

### Sample preparation

Recently extracted intact human incisors were stored at room temperature in distilled water until testing. The roots of the teeth were removed 2 mm apically to the cementoenamel junction using diamond discs, and the crowns were embedded in colorless self-cure acrylic-liquid mixture (Panacryl, Rubydent, Istanbul, Turkey). The enamel surfaces were ground flat using 400-grit silicon carbide abrasive paper and polished with 600 and 1200-grit aluminum oxide papers on a polishing machine (Mecapol P230, Presi, France), until a circular area of 10 mm diameter was exposed. The specimens were then subjected to color, surface microhardness and chemical composition analyses. For the standardization of the specimens, 170 teeth with the similar initial Vickers hardness values were selected for the study.

### Color measurements

The color distribution (L*, a* and b*) of each specimen was measured with a spectrophotometer (VITA Easy Shade, Vident, Brea, CA, USA). Measurements were taken at the middle third region of the specimens, which were repeated three times at each evaluation, and then their averages were calculated. The spectrophotometer was calibrated with a white reflectance standard according to the manufacturer's protocol before each measurement.

The overall color difference of specimen in each group was calculated by the following expressions ^12^ ^,^ ^13^: 

$$\Delta \text{E}={\left[{\left(\Delta \text{L}\right)}^{2}+{\left(\Delta \text{a}\right)}^{2}+{\left(\Delta \text{b}\right)}^{2}\right]}^{1/2}$$

### Microhardness evaluation

The microhardness values of the enamel surfaces were obtained with a microhardness tester (Shimadzu HMV/ 2000, Shimadzu Corporation, Kyoto, Japan). The Vickers hardness number (VHN) was determined by fitting a 50 kgf load into the diamond indenter, and by allowing the indenter to rest on the enamel surface for 30 s. Five indentations at the distance of 100 μm between were performed on each specimen to minimize interactions between neighboring marks. Then their average was calculated.

### Chemical composition analysis

Energy dispersive spectrometry (EDS) analyses were performed on the Scanning Electron Microscope (SEM) combined with EDS (Bruker Axs XFlash 3001 SDD-EDS, Cambridge, UK). Calcium (Ca), phosphorous (P) and fluoride (F) contents in mass percent were measured in standard mode from three peak spots *per* each specimen. The operating parameters were: 15 Kv accelerating voltage, 10 nA beam current, and 30-45-second counting times with a 10 mm working distance. Calcium and phosphorous contents were converted into Ca/P ratio for each specimen, and a range of Ca/P ratio were calculated.

### Bleaching and desensitizing procedures

The 13 specimens were treated at groups 1-5 according to the manufacturers’ instructions. The 13 specimens at groups 6-9 were treated with the bleaching agents mixed with desensitizers as described below:

*Group 1 (S):* 4-5 mini spoons Signal Professional White Now Powder [MC ITALIA srl, Lainate (MI), Italy] was well-mixed with 20-25 drops Signal Professional White Now Liquid [MC ITALIA srl, Lainate (MI), Italy] until a homogeneous paste was obtained, and it was applied all over the enamel surfaces as a 1 mm thick layer using a brush. Three 15-minute applications were made for each specimen, consecutively. After first and second applications, the bleaching agent was removed with cotton rolls, and, at the end of the third application period, the agent was rinsed off the enamel surface with running water.

*Group 2 (S&F):* The bleaching agent was prepared and applied as Group 1. After bleaching procedure, 0.5% fluoride containing gel Flor-Opal (F) (Ultradent Products, Inc. South Jordan, USA) was applied to enamel surfaces and left undisturbed for 4 min. Then the desensitizing gel was rinsed off with running water.

*Group 3 (S&TM):* After bleaching as Group 1, GC Tooth Mousse (TM) CPP-ACP paste (GC, Tokyo, Japan) was applied to enamel surfaces and left undisturbed for 4 min. Then CPP-ACP paste was rinsed off with running water.

*Group 4 (S&U):* After bleaching procedure, UltraEZ (Ultradent Products, Inc. Utah, USA) 3% potassium nitrate + 0.11 % fluoride (U) was applied to enamel surfaces for 4 min. The desensitizing agent was rinsed off with running water.

*Group 5 (S&SP):* Before bleaching procedure, Signal Professional SENSITIVE PHASE 1 (MC ITALIA srl, Lainate MI, Italy) 30% Nano Hydroxyapatite suspension (SP) was applied to enamel surfaces and left undisturbed for 2 min. The desensitizing agent was rinsed off with running water, and the bleaching agent was applied in the same manner. After bleaching, the application of SP was repeated.

*Group 6 (S-F mixture):* 1.5 mL of bleaching agent was mixed with 0.5 mL Flor-Opal (F) until a homogeneous paste was obtained. The S-F mixture was prepared freshly before each application period and applied 3 times for 15 min. After first and second applications, the S-F mixture was removed with cotton rolls and, at the end of the third application period; the mixture was rinsed off the enamel surface with running water.

*Group 7 (S-TM mixture):* 1.5 mL of bleaching agent was mixed with 0.5 mL GC Tooth Mousse CPP-ACP paste until a homogeneous paste was obtained and applied as Group 6.

*Group 8 (S-U mixture):* 1.5 mL of bleaching agent was mixed with 0.5 mL UltraEZ 3% potassium nitrate & 0.11% fluoride until a homogeneous paste was obtained and applied as Group 6.

*Group 9 (S-SP mixture):* 1.5 mL of bleaching agent was mixed with 0.5 mL Signal Professional SENSITIVE PHASE 1 30% Nano Hydroxyapatite suspension until a homogeneous paste was obtained and applied as Group 6.

After bleaching application and desensitizing procedures, samples were stored in freshly prepared artificial saliva for 14 days ^13^ . The artificial saliva solution was changed daily.

The composition of artificial saliva used was Na_3_PO_4_ (3.90 mM), NaCl (4.29 mM), KCl (17.98 mM), CaCl_2_ (1.10 mM), MgCl_2_ (0.08 mM), H_2_SO_4_ (0.50 mM), NaHCO_3_ (3.27 mM) and distilled water with the pH adjusted to 7.2. ^14^

Color, surface microhardness and chemical- composition measurements were repeated 1 and 14 days after bleaching procedures.

### Percentage of Microhardness Loss (PML)

PML was calculated using the following equations: ^15^

1. $$\text{PML}\left(\%\right)1\text{day after bleaching}=\left({\text{VHN}}_{\text{before}}-{\text{VHN}}_{\text{1day}}\right)/{\text{VHN}}_{\text{before}}$$
2. $$\text{PML}\left(\%\right)14\text{day after bleaching}=\left({\text{VHN}}_{\text{before}}-{\text{VHN}}_{\text{14day}}\right)/{\text{VHN}}_{\text{before}}$$

### Scanning Electron Microscopy (SEM) evaluation

One sample from each group was analyzed with SEM to examine the enamel morphology before bleaching and 1 and 14 days after bleaching. Specimens were cleaned with distilled water. SEM analyses performed on the EDS and SEM image combination were obtained at the time of EDS analysis, using a Zeiss EVO 50 EP SEM (Carl Zeiss, Cambridge, UK) without coating, as the vacuum conditions needed to sputter the enamel surfaces could result in deterioration. The specimens were left self-drying for 24 hours and no additional drying protocol was applied to the specimens for the SEM observations.

### Statistical analysis

Statistical analyses were performed with SPSS PASW, 15.0 software for Windows (SPSS Inc., Chicago, IL, USA). Normality of data distribution (Shapiro-Wilk test), as well as homogeneity of variance (Levene's test), was tested before statistical analysis. The Kolmogorov-Smirnoff test was applied to verify the data that were normally distributed. ANOVA test was used for homogeneous variances (microhardness values and ion contents in mass percent) (p<0.05), and the Welch ANOVA test was used for non-homogeneous variances (color difference) (p<0.05). Multiple comparisons of homogeneous and non-homogeneous variances were further evaluated using Tukey (p<0.05) and Dunnett T3 (p<0.05) tests, respectively.

## Results

Sample characteristics were similar for each group before test procedures for all the tested parameters. Table 1 shows the ΔE values of the groups. One day after bleaching, the highest ΔE was observed at Group 7 (S-TM mixture), which was significantly higher than Group 3 (S&TM), Group 5 (S&SP), Group 6 (S-F mixture), and Group 9 (S-SP mixture) (p<0.05). The ΔE value of Group 8 (S-U mixture) was the highest after 14 days and significantly higher than Group 2 (S&F), Group 3 (S&TM), Group 4 (S&SP), Group 5 (S&SP), and Group 6 (S-F mixture) (p<0.05).

**Table 1:** Means and standard deviations of color differences (ΔE) among the experimental groups 1 (B1) and 14 (B14) days after bleaching treatments

Groups	Evaluation Periods	
	B1	B14
G1 (S)	21.8 (6.8)^aA^ *	12 (12.1)^aB^
G2 (S&F)	24.4 (5.7)^aA^	10.9 (4.3)^bB^
G3 (S&TM)	20.2 (5.4)^bA^	7.2 (2.9)^bB^
G4 (S&U)	23.8 (6.6)^aA^	9.8 (3.5)^bB^
G5 (S&SP)	18.2 (5.1)^bA^	10.6 (4.1)^bB^
G6 (S-F mixture)	18.7 (4.7)^bA^	9.2 (3.2)^bB^
G7 (S-TM mixture)	28.4 (5.2)^aA^	14.7 (7.4)^aB^
G8 (S-U mixture)	24.5 (1.8)^aA^	17.3 (3.4)^aB^
G9 (S-SP mixture)	21.2 (6.6)^bA^	14.7 (3.8)^aB^

* Different letters (lowercase in the same column, uppercase in the row) indicate statistically significant differences (p<0.05)

Table 2 shows the Vickers Hardness Numbers (VHN). A decrease in microhardness was observed in all groups, except for Groups 6 and 7 after 1 day. The highest VHN was found in Group 6 (S-SP mixture), which was statistically similar to Group 7 (p>0.05). The microhardness of all groups increased in comparison with baseline after 14 days. All groups showed statistically similar microhardness 14 days after bleaching procedure (p>0.05). Table 3 shows the percentage of microhardness loss (PML). PML was observed in all groups, except for Groups 6 and 7 after 1 day. Group 1 showed the highest loss (p<0.05) followed by Groups 5, 4, 2, 3, 8 and 9. PML of Group 9 was very low (0.4%), which was different from the other groups (p<0.05). PML was not observed in all groups after 14 days. However, all groups exhibited a recovery. Groups 5, 6, 7, 8 and 9 showed better recovery than other groups (p<0.05). Group 8 showed the highest (23.1%) recovery rate after 14 days.

**Table 2:** Means and standard deviations of Vickers hardness number (VHN) among the experimental groups 1 (B1) and 14 (B14) days before and after bleaching treatments

Groups	Evaluation Periods		
	VHNBB	VHNB1	VHNB14
G1 (S)	417.9 (73.6)^aA^	325.6 (95.0)^aB^	420.4 (110.4)^aA^
G2 (S&F)	415.0 (76.9)^aA^	338.8 (68.9)^aB^	415.3 (65.8)^aA^
G3 (S&TM)	415.9 (57.6)^aA^	364.7 (71.9)^aB^	420.2 (98.0)^aA^
G4 (S&U)	419.3 (65.0)^aA^	340.0 (76.6)^aB^	425.2 (46.0)^aA^
G5 (S&SP)	412.9 (55.1)^aA^	328.5 (80.4)^aB^	483.2 (63.0)^aC^
G6 (S-F mixture)	408.4 (49.7)^aA^	522.4 (80.0)^bB^	493.1 (58.1)^aC^
G7 (S-TM mixture)	411.6 (49.3)^aA^	453.4 (117.8)^bB^	492.4 (72.1)^aC^
G8 (S-U mixture)	405.5 (60.2)^aA^	379.7 (88.9)^aB^	499.3 (67.8)^aC^
G9 (S-SP mixture)	413.0 (46.4)^aA^	411.5 (115.0)^aA^	461.3 (112.7)^aB^

Different letters with lowercase in the same column indicate statistically significant differences (p<0.05)

Different letters with uppercase in the same row indicate statistically significant differences (p<0.05)

**Table 3:** The percentage of microhardness loss (PML) (%) between 1 day before and after bleaching and 14 days before and after bleaching

Groups	PML (%)	
	One day before and after bleaching	Fourteen days before and after bleaching
G1 (S)	22.1	-0.6
G2 (S&F)	18.4	-0.1
G3 (S&TM)	12.3	-1
G4 (S&U)	18.9	-1.4
G5 (S&SP)	20.4	-17
G6 (S-F mixture)	-27.9	-20.7
G7 (S-TM mixture)	-10.2	-19.6
G8 (S-U mixture)	6.4	-23.1
G9 (S-SP mixture)	0.4	-11.7

Table 4 shows calcium (Ca), phosphorous (P) and fluoride (F) contents in mass percent and Ca/P ratios before bleaching and 1 and 14 days after bleaching. No significant change was observed after bleaching at Ca and P levels (p>0.05). F mass increased in Groups 2 and 6, 1 day after bleaching (p<0.05). No significant differences were found in Ca/P ratios 1 or 14 days after bleaching (p>0.05).

**Table 4:** Mean values and standard deviations of Ca, P and F (mass % contents) and Ca/P ratios

		Ca			P			F			Ca/P	
	BB	B1	B14	BB	B1	B14	BB	B1	B14	BB	B1	B14
G1 (S)	67.7	66.7	67.5	30.9	29.5	29.4	1.9	1.5	1.5	2.2	2.2	2.4
	(15)	(2.9)	(2.3)	(19)	-2	(3.6)	-3	-4	(13)	(0.1)	(0.2)	(0.4)
G2 (S&F)	68.2	67.7	68.4	30.2	29.7	29.7	0.8	15.6	1.6	2.3	2.2	2.4
	(15)	-9	(17)	-3	(3.8)	(13)	(19)	(12.7)^*^	(15)	(0.3)	(0.1)	(0.2)
G3 (S&TM)	67.6	68	68.9	30.6	30.8	30.1	1.8	2.8	2.1	2.2	2.2	2.4
	(2.5)	(3.6)	(15)	(19)	-3	(16)	(3.6)	(3.9)	(0.2)	(0.1)	(0.3)	(0.1)
G4 (S&U)	67.4	67.5	67.9	28.5	29.8	29.5	1.1	2.3	1.7	2.1	2.2	2.4
	(2.3)	(6.2)	(18)	(12)	(3.3)	(0.9)	(2.9)	(7.2)	(19)	(0.1)	(0.3)	(0.1)
G5 (S&SP)	65.8	67.1	68.9	30.6	31	30	1.6	1.9	1.7	2.2	2.2	2.3
	(4.1)	(2.3)	(2.9)	(18)	(16)	(19)	(5.8)	(3.1)	(3.9)	(0.1)	(0.1)	(0.2)
G6 (S-F mixture)	67	67.9	67.9	29.4	29.7	29.7	1.5	12.8	2.9	2.2	2.1	2.2
	(16)	-8	(3.5)	-1	(4.9)	(2.8)	(16)	(13.4)*	(4.8)	(0.1)	(0.4)	(0.2)
G7 (S-TM mixture)	67.1	67.3	69.4	30.7	30.7	30.7	1.2	2	1.2	2.1	2.2	2.4
	(18)	(2.9)	(19)	(15)	(13)	(12)	(2.8)	(3.5)	(16)	(0.1)	(0.1)	(0.2)
G8 (S-U mixture)	67.3	67.7	67.3	29.1	29.1	28.9	1.3	1.5	1.8	2.2	2.2	2.4
	(2.1)	(2.9)	(15)	-1	(16)	(11)	(2.6)	(3.7)	(18)	(0.1)	(0.1)	(0.1)
G9 (S-SP mixture)	67.6	68.5	70.6	31.4	30	30.1	1	1.9	2.3	2.2	2.3	2.4
	(18)	(3.5)	(15)	(11)	(18)	(13)	(2.4)	(4.2)	(12)	(0.1)	(0.2)	(0.2)

Abbreviations: Ca: calcium; P: phosphorus; F: fluoride; BB: Before bleaching; B1: 1 day after bleaching; B14: 14 days after bleaching

### Scanning Electron Microscopy (SEM) evaluation

**Figure 2:**
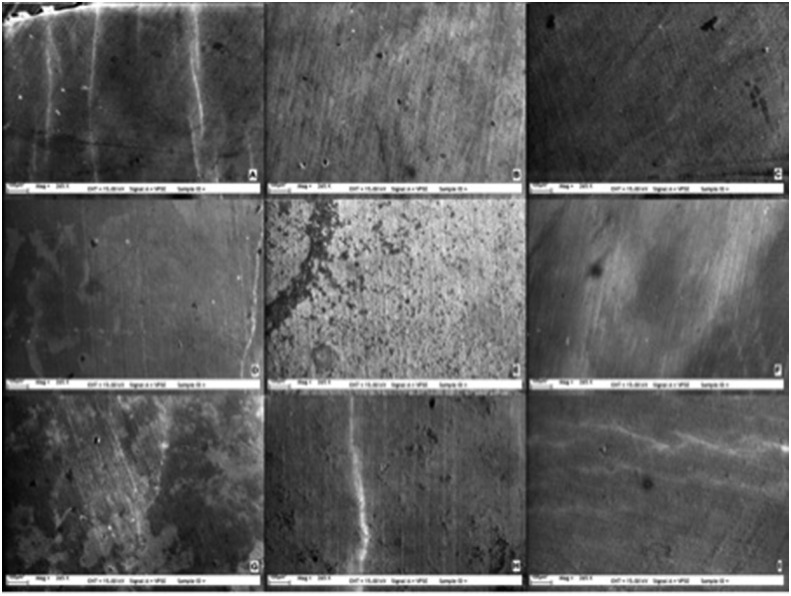
Scanning electron microscopy (SEM) photographs of Groups 1-9 (x265) before bleaching (A-I)

**Figure 3:**
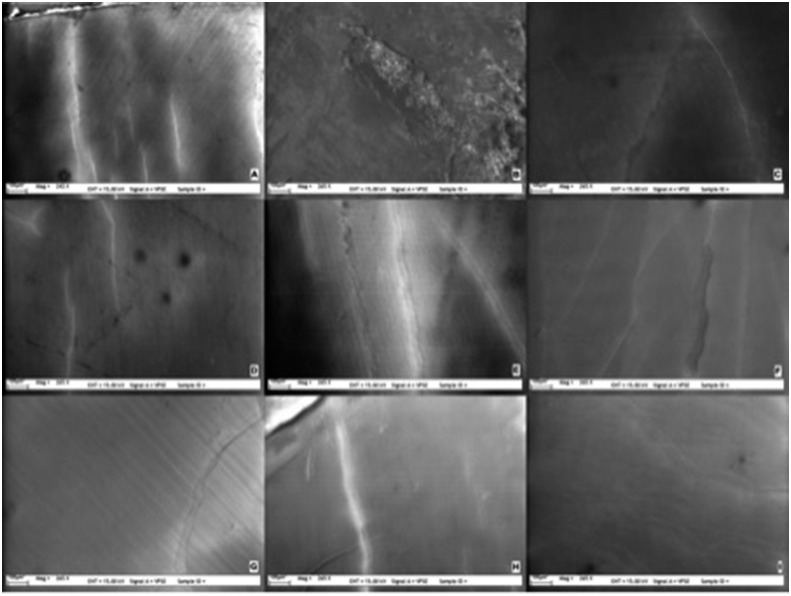
Scanning electron microscopy (SEM) photographs of Groups 1-9 (x265) 1 day after bleaching (A-I)

**Figure 4:**
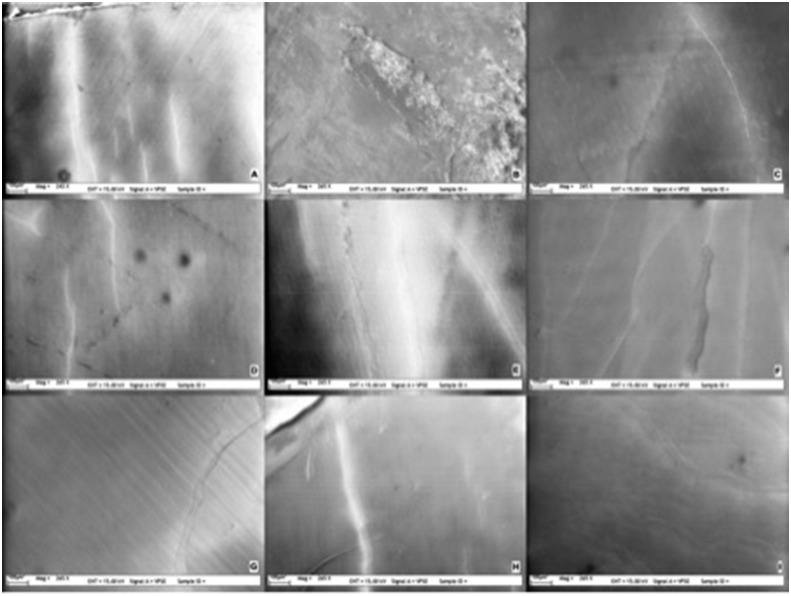
Scanning electron microscopy (SEM) photographs of Groups 1-9 (x265) 14 days after bleaching (A-I)

Surface characteristics of each group were similar to previous test procedures. SEM observations showed no deleterious effects for any of the groups neither 1 day nor 14 days after bleaching when compared with baseline ( Figure 2 - 4 ). No changes were observed on the enamel surfaces in the test groups at any evaluation time.

## Discussion

The most common side effect of all peroxide-based whitening procedures is tooth sensitivity, and efforts have been made to overcome the sensitivity caused by bleaching procedures. Bleaching with 35-38% hydrogen peroxides may alter enamel morphology, decrease microhardness and cause loss of hard tissue volume. ^8^ ^,^ ^16^ Thus, studies have been done to achieve a protocol, which may promote remineralization after bleaching and recover the microhardness loss and surface deterioration of enamel caused by bleaching.

The mixture of remineralizing and bleaching agents was capable of reducing sensitivity and recover or at least avoid alterations in the surface morphology of enamel. ^17^ Fluoride, potassium nitrate, ACP or n-HAP have been introduced in recent bleaching products to prevent either hypersensitivity or demineralization effect. ^18^ ^-^ ^20^ Moreover, these novel techniques did not decrease the bleaching potential of the peroxide. ^21^

In this study, the color change effect of the bleaching agent, both alone or combined with desensitizing agents (after bleaching or mixed with the bleaching gel), significantly decreased 14 days after the bleaching treatment when compared to 1 day after bleaching. This excludes any possible dehydration effect and represents the real effect in color change. Similarly, Zekonis, et al. ^22^ (2003) reported that the greatest values of color change were observed after bleaching, followed by a relapse 7 days after bleaching.

Application of fluoride and/or calcium was able to alter the microhardness loss in the post-treatment phase. Also, the addition of fluoride to the bleaching agent could positively affect the rehardening of bleached enamel, requiring shorter time for recovery compared with gels without fluoride. ^23^ In this study, enamel microhardness had a significant decrease after bleaching in all groups, except the specimens treated with the mixture of desensitizing agents containing fluoride or CPP-ACP. The specimens treated with the mixture of either fluoride or CPP-ACP showed the highest microhardness 1 day after the bleaching treatment.

Borges, et al. ^11^ (2011) evaluated the effect of CPP-ACP paste with a hydrogen peroxide agent on bleaching efficacy, level of tooth sensitivity, and alterations on enamel surface morphology. They reported that the use of a mixture of hydrogen peroxide office bleaching and CPP-ACP paste may reduce the tooth sensitivity and avoid morphological alterations on enamel after bleaching.

Potassium nitrate is one of the agents introduced in recent bleaching products to prevent hypersensitivity and demineralization effects by Chen, et al. ^8^ (2008); Grobler, et al. ^24^ (2009) showed the presence of potassium nitrate in the bleaching agents could not decrease microhardness in enamel. However, in this study, the potassium nitrate was not used alone but with fluoride.

Nano-hydroxyapatite (n-HAP) has currently gained wide acceptance in health sciences for being one of the most biocompatible and bioactive materials. Laboratory studies have shown microscopic surface enamel defects, associated with bleaching, could be repaired using a paste containing n-HAP crystals. ^25^ Because of its nano metric size, n-HAP can easily penetrate inside the dentinal tubules and enamel microcracks; thereby it promotes a reliable sealing for the tubules and microcracks, and it restores the microstructure and chemical composition of the tooth tissues.^18,26,27^ The n-HAP crystals are also very resistant to acidic challenges that routinely take place in the oral environment. ^28^

In this study, only 1 day after bleaching, 2 desensitizing agent groups showed an increase in initial microhardness values, all the groups were capable of maintaining microhardness values 14 days after bleaching.

The percentage of microhardness loss in this study ranged from 22.1 to 0.4 values. The highest loss was observed in the first group, in which no desensitizing agent was added, and the lowest loss was seen in the group in which the n-HAP was used as a mixture in the bleaching agent. The losses were remineralized 14 days after the bleaching treatment in all groups. This may be due to the extra effect of mineral concentration of artificial saliva.

In the daily routine of oral environment, acidic challenges lead to conditions for enamel remineralization, and previously demineralized enamel is known to be more susceptible to further remineralization. Bleaching agents may cause demineralization on the enamel, by which ionic changes are induced and mineral uptake is increased to replace the mineral loss during treatment. Therefore, the specimens were stored in artificial saliva to mimic the oral conditions. On the other hand, some studies suggest that saliva may partially lead to the replacement of the mineral loss caused by the bleaching treatment. ^29^ ^,^ ^30^ Basting, et al. ^31^ (2005) reported that immersion in a solution similar to human saliva for two weeks after bleaching may increase microhardness of bleached enamel. Similar to previous findings, saliva could present a reformative effect on the microhardness loss in this study.

Although the saliva is expected to remineralize the bleached enamel, some *in situ* studies have reported a decrease in microhardness on enamel immediately after bleaching treatments. ^15^ The microhardness loss could be related to mineral content loss caused by demineralization. The effects of bleaching agents on mineral loss of enamel and dentin are usually tested by microhardness studies for being directly related to the mineral content of the tooth. ^15^ ^,^ ^32^ For this reason, microhardness tests can be used both as a comparative measure of hardness changes and as a direct measure of mineral loss or gain as a consequence of demineralization and remineralization processes.

On the other hand, EDS determines the mineral content of dental hard tissues. The main advantage of this system is its capability to provide an accurate and non-destructive analysis of the specimens. ^33^ ^,^ ^34^ As a consequence, this method was used to evaluate the changes on mineral content of enamel in this study. The findings revealed that bleaching or desensitizing agents used after bleaching or as a mixture in bleaching agent did not affect Ca and P levels, but F levels increased in the groups treated with F-containing desensitizers after bleaching. The possible explanation for this increase may be the use of F-containing bleaching agent to prevent either sensitivity or demineralization during the bleaching treatment. However, controversial results exist on the topic, since no supporting evidence regarding the influence of F-containing bleaching gels on the demineralization has been documented. In an *in vitro* study, the bleaching agent either combined with F or Ca was insufficient to prevent the reduction in the surface microhardness of enamel. ^35^ Conversely, the addition of F in HP bleaching agent was shown to induce fluoridated HAP and Ca and F crystals on enamel surfaces when evaluated with an X-ray photoelectron spectroscopy. The remineralization processes of the demineralized tooth tissues are expedited by this mechanism. ^33^

Lee, et al. ^36^ (2006) reported a decrease in the Ca/P ratio of bleached bovine enamel after application of 30% HP. In contrast to their study, although 38% HP was used, the Ca/P ratios were not changed in this study.

With the limitation of this study, a specimen was examined *per* group under SEM and revealed no deleterious effects on enamel. The majority of the previous SEM studies that investigated the surface morphology following bleaching were in the same line with his study, reporting no significant changes. ^33^ ^,^ ^37^ On the other hand, some other studies have reported slight alterations on the enamel morphology with an increased number of pits, pores and erosion areas, which may also indicate demineralization and dissolution.^15,38,39^

Therefore, modifications of the bleaching gel did not influence the color change in this study. The desensitizing agents used after bleaching or as a mixture in the bleaching gel were capable of increasing the microhardness of enamel. Thus, the null hypothesis is accepted. However, more studies are needed to evaluate the effect of desensitizers used after bleaching or used in different modifications on dental hard tissues after extended periods of time.

## Conclusions

Within the experimental limitations of this *in vitro* study, the following conclusions could be drawn:

1. The use of desensitizing agents containing fluoride, CPP-ACP, potassium nitrate or n-HAP either after in-office bleaching or added to bleaching agent did not affect the color change.
2. Microhardness of enamel increased 14 days after in-office bleaching used with desensitizing agents.
3. Ca, P contents and Ca/P ratios did not change 14 days after bleaching. The F content increased in groups that contained only fluoride [G2 (S&F) and G6 (S-F mixture)] one day after bleaching.

## References

[1] Calazans FS, Dias KR, Miranda MS (2011). Modified technique for vital bleaching of teeth pigmented by amalgam: a case report. Oper Dent.

[2] Spalding M, Taveira LA, Assis GF (2003). Scanning electron microscopy study of dental enamel surface exposed to 35% hydrogen peroxide: alone, with saliva, and with 10% carbamide peroxide. J Esthet Restor Dent.

[3] American Dental Association (2010). Council on Scientific Affairs. Tooth whitening/bleaching: treatment considerations for dentists and their patients [Internet].

[4] Kihn PW (2007). Vital tooth whitening. Dent Clin North Am.

[5] Cartagena AF, Parreiras SO, Loguercio AD, Reis A, Campanha NH (2015). In-office bleaching effects on the pulp flow and tooth sensitivity - case series. Braz Oral Res.

[6] Sasaki RT, Catelan A, Bertoldo ES, Venancio PC, Groppo FC, Ambrosano GM (2015). Effect of 7.5% hydrogen peroxide containing remineralizing agents on hardness, color change, roughness and micromorphology of human enamel. Am J Dent.

[7] Nanjundasetty JK, Ashrafulla M (2016). Efficacy of desensitizing agents on postoperative sensitivity following an in-office vital tooth bleaching: a randomized controlled clinical trial. J Conserv Dent.

[8] Chen HP, Chang CH, Liu JK, Chuang SF, Yang JY (2008). Effect of fluoride containing bleaching agents on enamel surface properties. J Dent.

[9] Maleknejad F, Ameri H, Kianfar I (2012). Effect of intracoronal bleaching agents on ultrastructure and mineral content of dentin. J Conserv Dent.

[10] Pinheiro HB, Cardoso PE (2011). Influence of five home whitening gels and a remineralizing gel on the enamel and dentin ultrastructure and hardness. Am J Dent.

[11] Borges BC, Borges JS, Melo CD, Pinheiro IV, Santos AJ, Braz R (2011). Efficacy of a novel at-home bleaching technique with carbamide peroxides modified by CPP-ACP and its effect on the microhardness of bleached enamel. Oper Dent.

[12] Singh RD, Ram SM, Shetty O, Chand P, Yadav R (2010). Efficacy of casein phosphopeptide-amorphous calcium phosphate to prevent stain absorption on freshly bleached enamel: an *in vitro* study. J Conserv Dent.

[13] Kim YS, Kwon HK, Kim BI (2011). Effect of nano-carbonate apatite to prevent re-stain after dental bleaching *in vitro.*. J Dent.

[14] Jose P, Sanjeev K, Sekar M (2016). Effect of green and white tea pretreatment on remineralization of demineralized dentin by CPP-ACFP-an *in vitro* microhardness analysis. J Clin Diagn Res.

[15] Rodrigues JA, Marchi GM, Ambrosano GM, Heymann HO, Pimenta LA (2005). Microhardness evaluation of *in situ* vital bleaching on human dental enamel using a novel study design. Dent Mater.

[16] Shannon H, Spencer P, Gross K, Tira D (1993). Characterization of enamel exposed to 10% carbamide peroxide bleaching agents. Quintessence Int.

[17] Vasconcelos AA, Cunha AG, Borges BC, Vitoriano JO, Alves-Júnior C, Machado CT (2012). Enamel properties after tooth bleaching with hydrogen/carbamide peroxides in association with a CPP-ACP paste. Acta Odontol Scand.

[18] Ferraz LN, Vieira WF, Ambrosano GM, Giorgi MC, Aguiar FH, Lima DA (2018). Effect of different concentrations of nanohydroxyapatite on tooth bleaching effectiveness and enamel bond strength. Braz Dent Sci.

[19] Mellgren T, Qin T, Öhman-Mägi C, Zhang Y, Wu B, Xia W (2018). Calcium phosphate microspheres as a delivery vehicle for tooth- bleaching agents. J Dent Res.

[20] Kyaw KY, Otsuki M, Segarra MS, Tagami J (2018). Effect of sodium fluoride pretreatment on the efficacy of an in office bleaching agent: an *in vitro* study. Clin Exp Dent Res.

[21] Alqahtani MQ (2014). Tooth-bleaching procedures and their controversial effects: a literature review. Saudi Dent J.

[22] Zekonis R, Matis BA, Cochran MA, Al Shetri SE, Eckert GJ, Carlson TJ (2003). Clinical evaluation of in-office and at-home bleaching treatments. Oper Dent.

[23] Attin T, Betke H, Schippan F, Wiegand A (2007). Potential of fluoridated carbamide peroxide gels to support post-bleaching enamel rehardening. J Dent.

[24] Grobler SR, Majeed A, Moola MH (2009). Effect of various tooth-whitening products on enamel microhardness. SADJ.

[25] Minoux M, Serfaty R (2008). Vital tooth bleaching: biologic adverse effects - a review. Quintessence Int.

[26] Yan B, Yi J, Li Y, Chen Y, Shi Z (2013). Arginine-containing toothpastes for dentin hypersensitivity: systematic review and meta-analysis. Quintessence Int.

[27] Vano M, Derchi G, Barone A, Genovesi A, Covani U (2015). Tooth bleaching with hydrogen peroxide and nano-hydroxyapatite: a 9-month follow-up randomized clinical trial. Int J Dent Hyg.

[28] Mathew A, Reddy NV, Sugumaran DK, Peter J, Shameer M, Dauravu LM (2013). Acquired acid resistance of human enamel treated with laser (Er:YAG laser and Co_2_laser) and acidulated phosphate fluoride treatment: an *in vitro* atomic emission spectrometry analysis. Contemp Clin Dent.

[29] Dionysopoulos D, Koliniotou-Koumpia E, Tolidis K, Gerasimou P (2017). Effect of fluoride treatments on bleached enamel microhardness and surface morphology. Oral Health Prev Dent.

[30] Dey S, Pandey V, Kumar A, Awasthi N, Sahu A, Pujari SC (2016). *In vitro* comparison of impact of different bleaching agents on the microhardness of enamel. J Contemp Dent Pract.

[31] Basting RT, Rodrigues AL, Serra MC (2005). The effect of 10% carbamide peroxide, carbopol and/or glycerin on enamel and dentin microhardness. Oper Dent.

[32] Attin T, Schmidlin PR, Wegehaupt F, Wiegand A (2009). Influence of study design on the impact of bleaching agents on dental enamel microhardness: a review. Dent Mater.

[33] Cakir FY, Korkmaz Y, Firat E, Oztas SS, Gurgan S (2011). Chemical analysis of enamel and dentin following the application of three different at- home bleaching systems. Oper Dent.

[34] Llena C, Esteve I, Forner L (2018). Effects of in-office bleaching on human enamel and dentin. Morphological and mineral changes. Ann Anat.

[35] Furlan IS, Bridi EC, Amaral FL, França FM, Turssi CP, Basting RT (2017). Effect of high- or low-concentration bleaching agents containing calcium and/or fluoride on enamel microhardness. Gen Dent.

[36] Lee KH, Kim HI, Kim KH, Kwon YH (2006). Mineral loss from bovine enamel by a 30% hydrogen peroxide solution. J Oral Rehabil.

[37] Fatima N (2016). *In-vitro* comparative study of in-office and home bleaching agents on surface micro-morphology of enamel. J Coll Physicians Surg Pak.

[38] Pimenta-Dutra AC, Albuquerque RC, Morgan LS, Pereira GM, Nunes E, Horta MC (2017). Effect of bleaching agents on enamel surface of bovine teeth: a SEM study. J Clin Exp Dent.

[39] Coceska E, Gjorgievska E, Coleman NJ, Gabric D, Slipper IJ, Stevanovic M (2016). Enamel alteration following tooth bleaching and remineralization. J Microsc.

